# Music and Sound in Time Processing of Children with ADHD

**DOI:** 10.3389/fpsyt.2015.00127

**Published:** 2015-09-28

**Authors:** Luiz Rogério Jorgensen Carrer

**Affiliations:** ^1^Universidade Federal de São Paulo (UNIFESP), São Paulo, Brazil

**Keywords:** music, time processing, ADHD, music cognition, music therapy

## Abstract

ADHD involves cognitive and behavioral aspects with impairments in many environments of children and their families’ lives. Music, with its playful, spontaneous, affective, motivational, temporal, and rhythmic dimensions can be of great help for studying the aspects of time processing in ADHD. In this article, we studied time processing with simple sounds and music in children with ADHD with the hypothesis that children with ADHD have a different performance when compared with children with normal development in tasks of time estimation and production. The main objective was to develop sound and musical tasks to evaluate and correlate the performance of children with ADHD, with and without methylphenidate, compared to a control group with typical development. The study involved 36 participants of age 6–14 years, recruited at NANI-UNIFESP/SP, subdivided into three groups with 12 children in each. Data was collected through a musical keyboard using Logic Audio Software 9.0 on the computer that recorded the participant’s performance in the tasks. Tasks were divided into sections: spontaneous time production, time estimation with simple sounds, and time estimation with music. Results: (1) performance of ADHD groups in temporal estimation of simple sounds in short time intervals (30 ms) were statistically lower than that of control group (*p* < 0.05); (2) in the task comparing musical excerpts of the same duration (7 s), ADHD groups considered the tracks longer when the musical notes had longer durations, while in the control group, the duration was related to the density of musical notes in the track. The positive average performance observed in the three groups in most tasks perhaps indicates the possibility that music can, in some way, positively modulate the symptoms of inattention in ADHD.

## Introduction

ADHD involves cognitive and behavioral aspects that impact many environments of children and their families’ lives. Mainly, in social interaction and academic performance, there are frequent associations with psychiatric comorbidities (1). Difficulties in self-regulation under verbal commands, such as thinking before acting which affects non-verbal aspects of behavior and cognition, with difficulties in sequencing actions, executive modulation, reproduction, and motor coordination with rhythm patterns (2, 3). According to Castellanos and Proal (4), temporal dysfunction in ADHD is related to different neural networks that represent the baseline of brain physiology and are also important for cognitive processes like judgment, decision-making, and executive control. In a study by Sonuga-Barke and Castellanos (5), the authors suggested that ADHD could be considered a disorder of the default network. Some studies also indicate that methylphenidate can ease most of the time deficits and normalize the functioning of networks involved in ADHD, yielding to an improved performance in cognitive tasks (6, 7). In this sense, we think of music, as an eminently temporal and rhythmic art (8, 9) that displays lively, spontaneous, emotional, and motivational dimensions (10) that could possibly be of great help in studying aspects of time processing in children with ADHD. Our study investigated how the perception of time is related to the modulation of attention, executive, and inhibitory control of impulsive behaviors related to self-regulation, planning, and control of motor actions through sound and music. It is known that this depends on estimations and expectations of time as well as aspects linked to the handling of information in the working memory (11). Literature suggests that children who study or participate in musical activities have a better development in extra musical activities, such as learning languages, speed, and accuracy in reading and mathematics (12–15). Music, sound, and rhythm are stimuli that influence the perception of emotions, thoughts, and actions at the same time (16, 17). Therefore, music may contribute to the development of scientific research on neurodevelopment, memory, and cognitive functions (18–21). Time perception and representation are fundamental adaptive cognitive abilities that allow us to distinguish and organize sequences of events and actions, and predict when such events will occur in the future (22). Time estimation is an important adaptive ability and also facilitates to respond efficiently to sensorimotor demands (23, 24). Time processing is also a key issue for the neuroscience of cognition and behavior (25, 26). The way the internal time is self-regulated and standardized is critical for survival and for planning objectives and future goals (27–31). Certainly, perception of time affects our sleep, thoughts, memories, and consciousness, because we all have biological time patterns that regulate our body’s functions (9). The subjective sense of time can also be changed or modulated by motivational and affective involvement of the individual with the musical phenomenon, influencing attention while hearing or playing music in general and rhythms in particular. Emotions and attention can also guide reasoning for decision making (32, 33). In the traditional model, the brain has several temporal dimensions, which are neurologically processed by oscillators and timers for multiple time scales. The temporal processing of cognitive and motor functions occurs through different scales, in cycles of 24 h, a couple of hours, minutes, seconds, and milliseconds, and the accuracy varies in the range of milliseconds, which are also involved in the intrinsic properties of neural systems (26). Time processing in the scale of hundreds of milliseconds is essential for sensory motor synchronization, which takes place, for example, in finger tapping with an external stimulus, or playing a musical instrument in a band (34). In a neuropsychological point of view, time perception can be implicit (automatic and intuitive) and explicit (conscious and susceptible to cognitive manipulation), varying according to different contexts. According to Braitenberg (35), there are two subcortical brain structures related to temporal control: cerebellum and the basal ganglia. The explicit time perception involves the fronto-parietal network and time perception and motor coordination share common neural systems (36). There are several competing models to investigate time processing, and there are also several experimental paradigms for research in the literature (37). The ability to synchronize rhythmic activities concurrently to a temporal pattern can be performed either externally with a visual stimulus, played by an image on a screen, presented by a simple auditory stimulus using a isochronous pulse of a metronome, or it can even be induced by music, like seen in the present study. The time processing can be investigated through many theoretical models and research methods depending on the context (health and/or education). Previous research also focuses on the internal clock model (38) as an explanatory model of regulation of endogenous rhythms in the adult ADHD population. Studies with children found a prevalence of cognitive models, such as the attentional model by Zakay and Block (33), the motor behavior model (39), the motivational model (40, 41), and the executive functions and working memory model (42). These models may be considered as complementary, and not necessarily concurrent. There have been few studies about the role of time processing in the intrinsic modulation of executive and motivational functions in neuropsychology. Time estimation is the ability to estimate, anticipate, or perceive different intervals and durations of time. There are at least two types of tasks to investigate the notion of time estimation: prospective, related to predictability, in which the individual is alerted before the task that he/she should maintain the attention and then respond with a verbal or motor response; and in retrospective, related to surprise, in which the individual is not alerted before the estimation is to be held during the task (33). The prospective tests usually have two sounds of stimuli in different durations separated by an interval of time, the participant is then asked to respond verbally, comparing if the intervals were the same or different (43, 44). Time production is the ability to produce time durations according to predefined rules. In a spontaneous time task, the presenter asks the individual to produce a duration or period of time using a pulse or beat, made with a pencil on the table (tapping) or on a computer keyboard, consistently for a certain period of time (33, 45).

Time processing disorders are present in many neurodevelopmental disorders like ADHD, but also in degenerative neurological diseases, such as Parkinson’s and Alzheimer’s disease, manifesting symptoms of dysfunctional processing of time estimation, production, and reproduction tasks (46, 47). Children with ADHD have a lower performance in time estimation and production tasks (30), time reproduction (3, 28, 48–51), and temporal and motor skills when compared to those without ADHD (52–55). Among the models that explain such disorders in time processing, the attentional model predicts that when the stimuli exceed the capacity of a relevant path for the sensory events, higher demands are recruited from other cognitive functions, such as sustained attention and working memory (56, 57). In a recent study by Gomes et al. (58), interesting results indicated that children with ADHD are able to process temporal information automatically and that deficits presented through active discrimination paradigms occurred due to deficits in perception or subjective use of temporal information. It may be possible to establish a connection between authors, indicating the important role of executive functions, especially that of attention and working memory in temporal processing presented in ADHD. Toplak et al. (22) suggested that these deficits contributed to the lower performance of people with ADHD in the cognitive processes. In a study by Huang et al. (59), time discrimination played a role in predicting ADHD, and the time processing provided more information about the family history of the disorder. Rubia (6) also points out that an impulsive temporal behavior is characterized by low temporal tolerance and the abnormality in the time processing and temporal behavior is an aspect of ADHD. Radonovich and Mostofsky (2) suggested that there is a deficit in the use of temporal information in ADHD, resulting in deficits of working memory, and/or the use of cognitive strategies, rather than an issue around a central mechanism for time processing. In a study of Bauermeister et al. (3), results suggested that ADHD is associated with a specific deficit in the ability to reproduce, instead of estimating temporal duration, and this may be related to deficits in the inhibitory system and working memory. Yordanova et al. (60) suggested that the inattentive behavior of children is determined by multiple irregularities and there is a further guiding frequency fluctuation in the performances. Children with ADHD often overestimate time durations in the scale of seconds and their performance is also lower in time estimation in the range of milliseconds (61). There are few studies with musical parameters in the literature involving children with ADHD. For Benzon (62), it is important to investigate the contrast between musical stimuli and simple sound stimuli, because music can be explicitly organized in multiple temporal subdivisions between tens, hundreds of milliseconds and minutes with simultaneous activity in several hierarchically organized timescales. Taking this into account, our study sought to address both research methods making an adaptation of tasks found in the literature (references in Table 1).

**Table 1:** **Literature**.

Reference	Tasks	Stimulus	Results
(46)	Visual and auditive: discriminate durations	500 ms; 2, 3, 4, and 6 s	Visual: TDAH < control; auditive: no difference
(53)	Auditive: synchronized finger tapping	300 ms	TDAH: longer response time
(52)	Visual and auditive: synchronized finger tapping	167 ms, 200, 250, 286, 333, 400, 500 ms; 1 s	TDAH: difficulties in modulate response
(36)	Visual and auditive: duration discrimination	1 s	Discrimination limiar: TDAH > control
(54, 55)	Finger tapping	Spontaneous time	Speed: TDAH = control
			Variability: TDAH > control
(28)	Auditive: duration discrimination	400 ms	TDAH = control
(2)	Auditive: duration discrimination	550 ms; 4 s	Discrimination limiar:
			4 s: TDAH > control
			550 ms: TDAH = control
(65)	Finger tapping: synchronized and spontaneous	263, 500, 625, 750, 875 ms; 1 s	TDAH = control
(66)	Auditive and visual: synchronized finger tapping	400 ms; 1 s	Visual:
			Variability: TDAH > control
			Auditive: TDAH = control

## Materials and Methods

**Figure 1:**
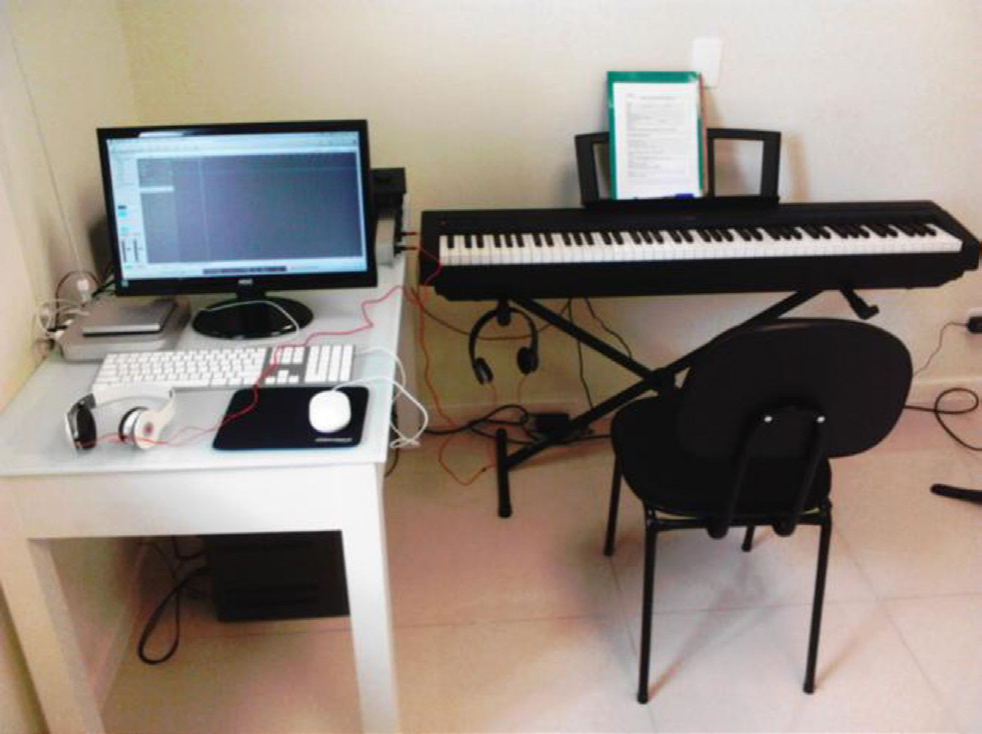
**Laboratory**.

The total sample consisted of 36 participants recruited by Núcleo de Atendimento Neuropsicológico Infantil Interdisciplinar (NANI) at UNIFESP, São Paulo, Brazil of ages 6–14, divided into three groups with 12 participants in each. The clinical sample consisted of 24 children with ADHD, subdivided into two subgroups: Group 1 – no medication (ADHD/NM) at least 7 days prior to the test; Group 2 – medication (ADHD/M) for at least 30 days prior to the performance of the test. Inclusion criteria considered for the study group: presence of at least six symptoms of inattention and/or hyperactivity/impulsivity in the DSM IV (SNAP Scale) (63), estimated IQ above 85 on the Weschler Child (WISC-III), normal hearing, absence of comorbidities in the symptom scales of child behavior check lists (CBCL) (*t*-score < 60) and good school performance. All children in the study were previously submitted to interdisciplinary evaluation that included medical and musical history, neurological, psychiatric, pedagogical, and neuropsychological assessment. Control group consisted of 12 children with typical development without symptoms of inattention or hyperactivity (less than two symptoms in SNAP Scale), IQ > 85 (WISC – III estimated), normal hearing and no health impairments. The control group was recruited with a letter to the school requesting permission from the board direction and the teachers. All participants and parents signed a consent form. The study excluded individuals with autism spectrum disorder, psychiatric comorbidities, and formal music study in order to avoid possible strategies of implementation and performance on tasks. Data was recorded through evaluation sheets, and the performance of participants was recorded in the computer through the keyboard connected to a digital interface for musical instruments (Avid MC-400). Data analysis was performed using SPSS 20.0 (Statistical Package for Social Sciences) (64) with non-parametric tests with 95% significance level (*p* < 0.05). The study was held in an appropriate room with all the necessary instruments and a comfortable and quiet environment to allow for the most accurate results. Figure 1 shows the laboratory.

### Procedures

The Ethics Committee of the Federal University of São Paulo, who had prior knowledge about the topic and research steps for participants and their families, approved this research. The protocol contained temporal estimation and temporal production tasks with many levels that assessed aspects of time perception and time expression through sensory estimations and motor time production activities. The tasks were conducted on the computer with the participant using the musical keyboard to perform the task. The tasks were performed with headphones to mitigate the influence of external stimuli. The headphones also ensured auditory perception of better sound quality, speed, and efficiency in the transmission of sound codes. The hearing level of the tasks was set to 60 dB. Sound stimuli and music were recorded in the laboratory with LogicAudio-9 software for music composition, editing, and audio recording; Yamaha electric piano; AKG microphone AK-400; AK-K141 headphones; an iMAC computer; and an audio and MIDI interface – Avid-MC400 (Figure 1).

### Tasks

The sound and musical tasks were divided in Task 1 – spontaneous time was based on a spontaneous time test by Mira Stambak (45) and adopted to be performed and registered on the computer. The participant was instructed to tap a regular freeform beat. The beat played by the participant was recorded on the computer and the analysis considered the performance of the three groups according to the dispersion from median 10, over 10 s (>10 s – Slow) and below 10 s (<10 s – Fast) to perform 21 beats. Task 2 – time estimation with simple sounds: twenty pairs of simple sound with different durations and separated by short intervals with silence. The instructor provided the participant with the rules of the task and then played the prerecorded instructions that contained three pairs of stimuli for training and adaptation. The participant then heard each pair of sounds and compared the durations by responding verbally if the stimuli were the same or different. Stimuli contained periods between 500 ms and 1 s, with the differences in duration varying from 0 ms (no difference), 20 ms (2%), 30 ms (3%), 50 ms (5%), 70 ms (7%), 100 ms (10%), 150 ms (15%), 200 ms (20%), 300 ms (30%), and randomly arranged. The number of correct answers were analyzed and compared between groups, which also showed minimum differences in the durations detected. Task 3 – time estimation with music: eight musical tracks, with each track containing two different songs, but with the same length of 7 s, separated by a 3-s interval of silence. Within each song, there were ranges with different densities of musical notes (number of musical notes/sound events) and was recorded on the keyboard using piano timbre. The tracks were organized and presented to the participant as follows: Track 1/music: “a” (7 s) and music “b” (7 s), and so on to the next tracks. The participants answered if the songs’ duration were the same or different. In the case, the participant answered that the songs were different the instructor asked: which is the longest song, “a” or “b”? Then, the subjective time perception regarding the lengths and densities of different songs was evaluated searching for individual differences in the time perception.

## Results

### Task 1

#### Spontaneous Time

The average spontaneous time of ADHD group without medication was faster (8.7 s). Compared to the other two groups, this difference was not statistically significant. However, participants in Group ADHD without medication conducted the beats in <10 s (Fast), while Group ADHD under medication played 21 beats in >10 s (Slow). In the control group, the performance was more balanced as shown in Table 2.

**Table 2:** **Spontaneous time**.

	Time (s)			
Group	0–10	>10	Median	SD
TDAH/NM	9	3	8,779	2.40
TDAH/M	4	8	10,032	2.48
Control	7	5	9,362	2.40
Total	20	16	9,391	2.41

*p > 0.05*.

### Task 2

#### Time Estimation with Simple Sounds

A statistically significant difference was observed when the three groups performed the stimulus 18, after a minute (*p* < 0.05), where the difference in the duration of the two stimuli was only 3% (30 ms).

### Task 3

#### Time Estimation with Music

**Figure 2:**
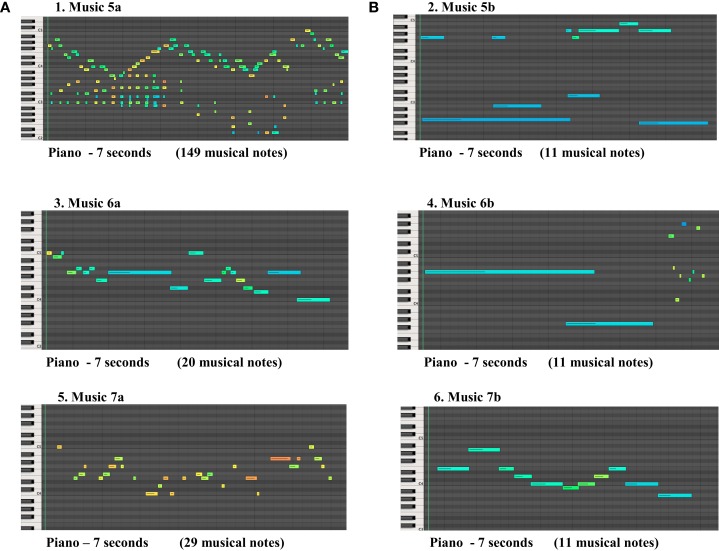
**The figures above present three pairs of musical stimuli (A,B) showing the musical notes perceived as longer by ADHD group in the right (B), in contrast with musical notes perceived as longer by the control group in the left (A)**.

There was a statistically significant difference between the control group and ADHD groups in Track 7 (*p* < 0.05), where all participants in the control group chose music “a”, as longer, and ADHD groups chose music “b” as longer. It was observed that the choice of the participants regarding the longer music was well balanced, when the density of musical notes in the music was higher, as shown in Figure 2.

## Discussion

According to Mithen (67), the spontaneous time (Task 1) is part of our automatic and implicit behavior with little involvement in cognition. The fact that this was preserved in ADHD may suggest that the implicit time function has not changed. We also noted that the results corroborate the findings of Rubia et al. (55) and Tiffin-Richards et al. (65), where there were no statistically significant differences between the ADHD and control groups in tasks of finger tapping in spontaneous time. There is much heterogeneity in the evaluation of spontaneous time in the literature, but one of the biggest differences is the modality of the stimulus. This requires different methods of analysis and interpretation of data depending on the hypotheses and research models. A larger sample is needed to better elucidate whether these performance differences were relevant for the phenotype of ADHD. The results of Task 2 (time estimation with simple sounds) corroborate the studies by Breier et al. (43), where the group with ADHD achieved a lower performance compared to the control group in time estimation tasks with auditory stimuli with only minor differences of 30 ms in length. In a study of Toplak and Tannock (66), the performance of the ADHD group was also lower in tests with stimuli containing periods between 200 ms and 1 s. Brown and Vickers (44) found no differences between groups ADHD and control in time estimation tasks using visual stimuli. Radonovich and Mostofsky (2) found differences between the control group and ADHD in auditory tasks lasting 4 s and, similar to our study, no differences between the groups in duration of 550 ms. Barkley et al. (27) and Meaux and Chelonis (49) found no differences between ADHD and control groups in verbal time estimation tasks, with periods where the difference was between 2 s and 60 s. Our results may indicate that the temporal processing of duration in the scale of hundreds of seconds would be preserved in the ADHD group due to the automated processing of temporal information (11). Due to fast integration of auditory and sensory motor systems, and high cognitive level processes which shall include music (68), we realized the need to expand the studies about time processing with different types of stimuli and concurrent tasks (dual tasks). The results of Task 3 showed that in our study, music with more musical notes at the same period of time (7 s) was perceived by most individuals of the three groups as longer. This may suggest that there is indeed a high attentional demand on counting sound events causing the individual to perceive time as longer depending on the quantity of events. Similar data were found in studies from Droit-Volet et al. (41). The same effect can be obtained in other contexts in which the individual undergoes stressful situations where attention is focused on the timing, causing the perceived subjective time to be perceived as longer than the time clocked (69). Emotions also play an important role in time perception (40, 70). Our results corroborate studies by Boltz (71) with musical stimulation, where time intervals of equal duration are filled with more or fewer elements. In another study of Lejeune (72), the author discusses the theories of temporal processing with the conclusion that the attentional model and reference memory placed by Zakay (73, 74) and Grondin (75), would be more appropriate for the paradigms in time estimation, by involving various aspects of attention and the memory combined. The author argues that it is not necessary to complement the model with the internal clock of Mattel and Meck (76). The Group ADHD/NM confirms the tendency to overestimate stimuli that require more attentional capacity, corroborating studies of Barkley (77), or when the stimuli exceed the echoic memory (68). Thus, some results of our study may support the role of the attentional window model proposed by Zakay and Block (33) and Zakay (78), where the time processing is flexible and susceptible to cognitive and pharmacological manipulation, not hard or fixed, as the dependent implicit and endogenous temporal cyclers in the internal clock model (76). Noulhiane et al. (79) and Droit-Volet and Gil (70) reported in their studies the influence of factors, such as emotions to the perception and the temporal information processing. The influence of context, the relationship with the modality of stimulation, empathy with the environment, with the music, and motivation may have positively influenced the performance and can be better evaluated in future studies, determining, for example, the affective valence that children attribute to musical tasks. The positive average performance observed in the three groups, in most sound and musical tasks perhaps indicates the possibility that the music can, in a certain way, positively modulate the symptoms of inattention. Research can also be developed in order to minimize social, pedagogical, and educational negative impacts of these disorders through the mediation of music.

## Conflict of Interest Statement

The author declares that the research was conducted in the absence of any commercial or financial relationships that could be construed as a potential conflict of interest.
